# Comparing High-Intensity Interval Training (HIIT) and Continuous Training on Apelin, APJ, NO, and Cardiotrophin-1 in Cardiac Tissue of Diabetic Rats

**DOI:** 10.1155/2020/1472514

**Published:** 2020-08-27

**Authors:** Mostafa Sabouri, Javad Norouzi, Yashar Zarei, Mojtaba Hassani Sangani, Babak Hooshmand Moghadam

**Affiliations:** ^1^Department of Exercise Physiology & Health Science, University of Tehran, Tehran, Iran; ^2^Oxygen Sports Medical Center, Tehran, Iran

## Abstract

**Background and Aims:**

Exercise activity is an important method for managing type 2 diabetes. This investigation examined the HIIT and continuous training on *apelin*, *APJ receptor*, *NO*, and *cardiotrophin-1* in the cardiac tissue of diabetic rats.

**Methods:**

The animals were categorized into 3 groups of HIIT, continuous (CO), and control (C) (all animals were sacrificed immediately and 2 days after exercise training period). Rats underwent the treadmill exercise program either HIIT (12 bouts at 90–95% of VO_2_ max with 60 s rest at 50% of VO_2_ max) or CO (60–65% VO_2_ max for 40 min). Protocols performed 5 days per week for 8 weeks. Apelin, APJ receptor, NO, and cardiotrophin-1 protein expressions were measured using the Western blotting method in the left ventricle.

**Results:**

Immediately after HIIT and CO exercise protocols, *apelin* and *CT-1* protein showed a significant difference in contrast by the C-0 group (*p* < 0.01). However, *NO* values were substantially higher in HIIT-0 compared to C-0 and CO-0 groups rats (*p* < 0.01). After two days of exercise protocols, *apelin* and *NO* protein showed a significant increase in HIIT and CO groups in contrast to the C animals (*p* < 0.01). Moreover, *APJ* and *CT-1* protein significantly upregulated in CO-2 and HIIT-2 compared to the other groups (*p* < 0.01).

**Conclusions:**

This study indicates that exercise training, despite the type, is an efficient method to modify *apelin*, *APJ receptor*, *NO*, and *cardiotrophin-1* values in animals with type 2 diabetes.

## 1. Introduction

Type 2 diabetes mellitus (T2DM) is distinguished as a metabolic dysfunction described by high-blood glucose in relation to comparative insulin deficiency and resistance of insulin [1]. T2DM is an extremely widespread disease and it is suggested that in 2040, about 642 million persons will suffer from it [2]. Additionally, it has been shown that diabetes is linked with crucial complication and health problems, like cardiovascular disease, nephropathy, and muscle atrophy [3].

Apelin, identified as an adipokine, is potentially upregulated by insulin, and increment of its values has been detected in obesity, insulin resistance, and T2DM in both humans and animals [4]. Moreover, apelin applies its function through activating the APJ, angiotensin receptor-related G protein-coupled receptor [5]. Various tissues such as muscles, adipose, lung, and cardiovascular system expressed apelin and APJ [6, 7]. However, increasing evidence emphasizes that apelin plays a useful role in metabolic dysfunction and has antiobesity and antidiabetic functions [8, 9]. In this regard, several animal types of research have been shown the effective role of apelin-APJ signaling in obese/diabetic conditions [10]. Furthermore, the dysfunction of the apelin-APJ system in diabetes and cardiovascular disorders induced reduction and increment of vasodilatation and vasoconstriction responses, respectively [11–13]. Apelin peptides through stimulation the release of nitric oxide (NO) cause endothelium-dependent vasorelaxation; by contrast, endothelial NO synthase (eNOS) inhibitor could almost eliminate this effect [12, 14]. Therefore, it seems that apelin may have a vasodilatation role through activation of NO pathway. Cardiotrophin-1 (CT-1) belongs to the interleukin-6 (IL-6) of cytokines that contributed in energy metabolism and induced hypertrophy in cardiomyocytes [15, 16]. Interestingly, the lack of CT-1 in animals improved insulin resistance, obesity, and dyslipidemia; by contrast, long-term administration of CT-1 diminished bodyweight and ameliorate insulin resistance in obese mice [15, 17]. Although CT-1 might contribute to insulin sensitivity [15] and improving metabolic disorders [18], its function in the metabolism of glucose and lipid is still unclear.

In addition, regular exercise training has been recognized as effective intervention methods in order to prevent and manage metabolic disorders such as diabetes. Exercise training by stimulating muscle glucose uptake may maintain chronic glycemic control [19] in T2DM and beneficial in decreasing diabetic complications [20]. Previous researches have shown that exercise training could enhance apelin and APJ values in obese individuals and animals [4, 21]. Moreover, in one study, CT-1 levels were substantially different between trained and untrained subjects [22]. However, after 8 to 10% weight loss, CT-1 values have not shown significant change [23]. Nevertheless, the level of participation of diabetics in exercise activities remains low due to time deficiency and difficulty in exercise programs [24, 25]. In this regard, high-intensity interval training (HIIT) is a choice that may by a shorter duration could provide the same or larger benefit than continuous training by moderate exercise for metabolic health [26]. Prior researches have found different results in comparison to HIIT and continuous training T2D [27, 28]. Furthermore, the researches about the comparison of HIIT and CO training on apelin/APJ system, NO, and CT-1 in diabetic rats are limited. Thus, we investigate the effect of HIIT and CO on apelin, APJ, NO, and, CT-1 in the cardiac tissue of diabetic rats.

## 2. Procedures

### 2.1. Animals

Male Wistar rats (*n* = 40; age = 12 weeks and weight range of 220-240 g) were obtained from the Animals Center of Pasture Institute of Iran. Animals were housed in the Exercise Physiology Department, at the University of Tehran (12/12 h light/dark cycle at 22°C and RH = 45%). Three to five rats were in glass cages with a lid and dimensions of 25 × 27 × 43 cm, which have free access to standard water and food. After 2 weeks of acclimatization period to the new environment, 4 rats were selected as the pilot group and diabetes was make through injection of streptozotocin (STZ), and they were examined for preliminary studies and the ability to perform HIIT and continuous training protocol. After a pilot study, animals were randomly separated into three groups: HIIT (HIIT), continuous training (CO), and control group (C).

### 2.2. Induced Diabetes

Following acclimatization time (one week), diabetes was induced through intraperitoneal injection of streptozotocin (STZ) (50 mg/kg), solved in citrate buffer (pH 4.5). Two days after STZ injection, fasting blood glucose was determined via a small nick in the tail in order to confirm the diabetes animal model. Diabetes rats represented blood glucose levels up to 300 mg/dL.

### 2.3. Exercise Training (HIIT and CO Protocols)

After STZ-induced diabetes and familiarization week to treadmill exercise (10 min, speed 10 m/min, 5 days/week), animals performed each of HIIT or CO protocols. The HIIT training contains 12 bouts of 1 min running with 90-95% of VO_2_ max and 60 s rest at 50% of VO_2_ max with 20 m/min speed in the beginning week, which regularly elevated to 30 m/min in the 8th week. Animals in the CO carry out 40 min of treadmill running training with a constant speed of 60–65% of VO_2_ max throughout the whole training period [29]. It should be noted that control animals have not performed any exercise training. Moreover, 5 min was considered for warmup and cool down. To motivate the exercise animals to run continuously, sessions of mild shock (intensity = 0.5 mA), which do not create stress on rats, were utilized.

According to previous studies, exercise capacity has been measured before the exercise training [2, 30]. In short, the rats were running at 6 m/min intensity on a graded treadmill at 15° inclinations. Then, the speed increased every 3 minutes by 3 m/min. This increase continued until the rats became exhausted. The whole distance traveled by rats was considered as exercise capacity.

### 2.4. Tissue Preparation

Immediately and after 2 days of the last training session, animals were anesthetized (ketamine (90 mg/kg) and xylazine (10 mg/kg)), and the hearts were obtained immediately, frozen in liquid nitrogen, and kept at −80°C for next analysis [31].

### 2.5. Western Blotting

The protein content of apelin, APJ, NO, and cardiotrophin-1 was measured using western blotting Tanique as with previously performed by other researches [2, 3, 32]. The frozen cardiac tissues were homogenized in 1 ml of lysis buffer (50 mmol/L Tris–HCl pH 7.4, 1% NP-40, 0.25% sodium deoxycholate, 150 mmol/L NaCl, 1 mmol/L EDTA, 1 mmol/L phenylmethylsulfonyl fluoride, 1 mmol/L Na3VO4, and 1 mmol/L NaF) and incubated at 4°C for 30 min, followed by centrifugation at 10,000*g* at 4°C for 20 min. The lysates were collected, and the protein concentration was determined using the Bradford Assay kit. Equal amounts of protein were loaded on 10% polyacrylamide and then transferred to polyvinylidene difluoride membrane (Roche, UK). Next, the membranes were incubated with primary antibodies overnight at 4°C, then secondary antibody (anti-goat or anti-rabbit IgG conjugated to horseradish peroxidase). Enhanced chemiluminescence (ECL) detection kit was used for the detection of antigen-antibody complexes. Images were quantified using ImageJ 1.63 software.

### 2.6. Statistical Analysis

Statistical data were shown as mean ± SD. One-way ANOVA has been used to detected differences among groups with the SPSS software (21.0 version). *p* ≤ 0.01 was considered as a significant statistical level.

## 3. Result

Previous reports suggest that microinjection of streptozotocin-induced diabetes in the rat [1, 3] two days after surgery showed that blood glucose levels are more than 300 mg/dl in all animals (Table 1). Additionally, bodyweight represented in Table 1.

### 3.1. Apelin and APJ Protein Expression in HIIT and CO Groups in the Left Ventricle Tissues in Diabetic Rats

**Figure 1:**
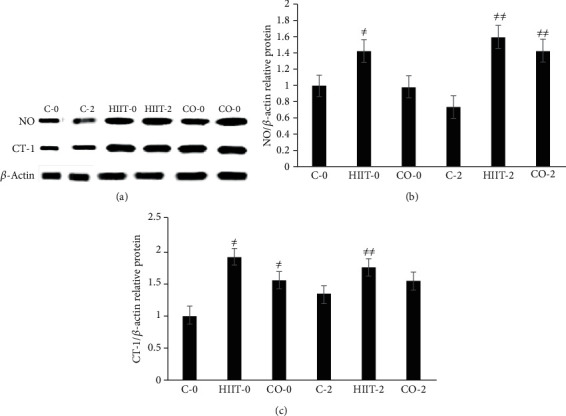
The impact of HIIT and CO exercise training on the (a) protein expressions of NO and CT-1 in the cardiac tissue of diabetic rats, (b) protein expression of NO, and (c) CT-1 proteins in different groups. The amount of rat *β*-actin used for normalizing the relative level of each protein, which expressed as the mean ± SEM (*n* = 3/group). ^≠^*p* < 0.01 vs. control (C-0) group; ^≠≠^*p* < 0.01 vs. C-2 group animals.

**Figure 2:**
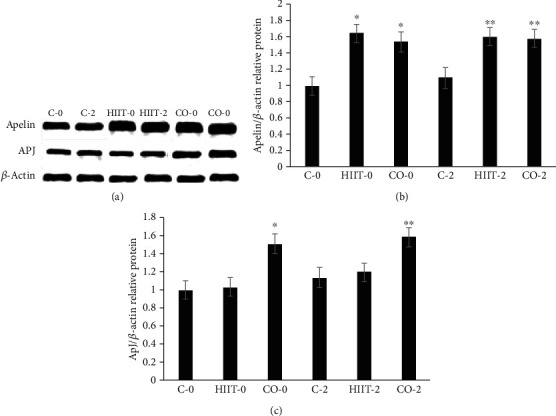
APJ and apelin protein values in the cardiac tissue (a–c). The amount of rat *β*-actin used for normalizing the relative level of each protein. Values are mean ± SD (*n* = 3, for all groups); ∗*p* < 0.01 vs. control (C-0) group; ∗∗*p* < 0.01 vs. C-2 group animals.

As presented in Figure 1(b), there is a considerable improvement in HIIT-0, CO-0, and HIIT-2, CO-2 groups compared to C-0 and C-2 groups in apelin protein content (*p* < 0.001). Also, the results showed that no substantial difference has been found between apelin protein in HIIT and CO groups immediately and 2 days after the last exercise session (*p* ≥ 0.01). Moreover, although HIIT and CO protocols showed an increase in APJ protein expression compared control group, nevertheless only CO-0 and CO-2 showed significant difference compared to the HIIT-0, HIIT-2, C-0, and C-2 groups (*p* < 0.01, Figure 2(c)).

### 3.2. NO and CT-1 Protein Expression in HIIT and CO Groups in the Left Ventricle Tissues in Diabetic Rats

The data demonstrate that NO protein in the HIIT-0 group was considerably higher than in C-0 and CO-0 group rats (*p* < 0.01, Figure 1(b)), while there are no statistical differences of NO protein among CO-0 and HIIT-0 rats. Furthermore, both HIIT and CO exercise training significantly improved NO levels compared to C diabetic rat group after 2 days (*p* < 0.01, Figure 1(b)). Additionally, CT-1 protein was considerably increased in HIIT-0 and CO-0 groups compared to the C-0 group (*p* < 0.01, Figure 1(c)), whereas CT-1 protein content was significantly elevated in HIIT-2 in comparison to compared C-2 and CO-2 (*p* < 0.01, Figure 1(c)). No differences in changes of CT-1 protein were found among HIIT and CO rats immediately and after 2 days of the last exercise session. Moreover, it should be noted that HIIT had a greater effect on CT-1 than CO in diabetic rats.

## 4. Discussion

T2DM, as a metabolic disorder, has shown to damage different organs through micro and macrovascular injuries as well as compromising the life of quality of the diabetes population [32]. Moreover, regular exercise training is considered as a nonpharmacological method in managing and preventing of type II diabetes. In this regard, we used the T2DM animal model to investigate the effect of HIIT and CO protocols on apelin, APJ, NO, and cardiotrophin-1 in the cardiac tissue.

Our results found that CO and HIIT upregulated apelin protein values in left cardiac ventricular in diabetic animals. Additionally, a substantial development was observed in APJ protein in CO-0 and CO-2 in diabetic rats. Prior researches reported that apelin might relate to T2DM [33] and might contribute to insulin sensitivity and metabolism of glucose [33, 34]. Moreover, apelin levels in diabetes patients have a direct relationship with the amount of physical activity [35]. In line with our results, in obese mice, exercise training in hypoxic conditions stimulates the expression of apelin/APJ in the skeletal muscle [21]. In another study, apelin mRNA in the myocardial and aorta has been shown to be increased following aerobic training in the hypertensive animals [36]. Apelin stimulation by exercise training has not yet been fully clarified. Although exercise appears to stimulate a polybeneficial adaptation in the cardiovascular system, growth in apelin seems to be a response to exercise adaptation in the cardiovascular system [36]. Moreover, according to previous studies, it seems that exercise training through insulin signaling pathway, PI3K [37], and AKT phosphorylation [38] induced skeletal muscle apelin expression in T2DM [38, 39].

Furthermore, many types of research have found a reduction of apelin values in response to exercise training in the T2DM state. In this regard, in an animal's model, long-term aerobic training alleviated apelin/APJ in the fat and muscle tissues [40]. Sheibani et al. [41] and Krist et al. [42] reported that exercise training could diminish the apelin value and apelin mRNA in plasma and fat tissue, respectively, in obese individuals and impaired glucose-tolerant patients. It seems that exercise training has a varied impact on apelin/APJ expression in various organs, like the skeletal muscle, adipose, and cardiac, which indicates that distinct signaling pathways control apelin and APJ expression in different organs. Therefore, more researches are needed to better comprehend exercise training's impacts on apelin/APJ production in various organs in diabetic people.

Disruption in the apelin/APJ system resulted to decrement vasodilatation and improved vasoconstriction responses described in diabetes and cardiovascular dysfunctions [11, 13, 43]. Apelin triggering the release of nitric oxide (NO), which not only plays an essential role in vasomotricity but also in skeletal muscle glucose uptake. A number of studies showed NO availability in both animal and human type 2 diabetic subjects [44, 45]. Our data showed that both HIIT and CO protocols induced increases in myocardial NO protein in diabetes rats as previous researches found. In this regard, 7 weeks of exercise training reversed endothelial impairment through the increment of NO production in the T2DM animal model [46]. Furthermore, Laher et al. [47, 48] indicated that long-term wheel running improved endothelial function in diabetic mice, although insulin and blood glucose have not changed. According to the current study and previous studies, exercise seems to act an essential role in ameliorating endothelial function, especially in diabetics. An increase in nitric oxide has led to improved glucose uptake and the sensitivity to insulin in diabetics. Besides, it seems that the apelin/eNOS axis has been implicated in processes such as glucose uptake [38] and vascular functions [13, 14], and not surprising that this axis dysfunction is associated with diabetes. The exact mechanism of the increase in nitric oxide through apelin is not clear. However, this increase appears to be due to phosphorylation of the PI3K/Akt/eNOS pathway [49]. The present study also showed that intense exercise had a greater effect on increasing nitric oxide in diabetic mice, which indicates the importance of the role of exercise intensity. In line with our results, Chavanelle et al. reported intense exercise training to have a greater impact on skeletal muscle Glut4 content and blood glucose in both healthy and obese mice [50].

Moreover, we present in that CT-1 protein value improved substantially in training groups (HIIT-0 and CO-0) after 8 weeks in rats with diabetes. Furthermore, CT-1 protein expression was significantly increased only in HIIT-2 compared to C-2 and CO-2. It has been suggested that CT-1 induced glucose-stimulated insulin secretion [51]. Moreover, the injection of CT-1 reversed insulin resistance in CT-1-deficient mice [15, 52], which considers the prominent role of CT-1 in the treatment of metabolic disorders and obesity. Another research showed that CT-1 values were substantially lower for overweight and obese subjects than those with normal weight [53]. In addition, although many types of research have been performed on the role of exercise and diabetes, our study seems to be the first to study that compares the role of HIIT and CO exercise on CT-1 in the heart muscle of diabetic mice. However, one study found that CT-1 values in athletes showed substantially higher in contrast to the control group [22]. Another study reported no considerable difference in CT-1 levels after 8 to 10 percent weight loss [23]. One mechanism that may be involved in CT-1's insulin-sensitizing effects is its ability to oxidize FFA, as suggested by high levels of FFA increase insulin resistance in major organs of the body [54]. In this regard, the decrement of lipolytic genes and the lipolytic response has been found in CT-1 null mice, which stimulates body fat enhancement and insulin resistance [55, 56]. On the other hand, due to the increase in CT-1 in individuals with metabolic syndrome and association with hyperglycemia, CT-1 appears to induce insulin resistance [18, 57]. In contrast with this point, both HIIT and CO protocols increased CT-1 content that follows with an improvement of blood glucose levels in diabetics' animals. Moreover, it should be noted that HIIT had a greater effect on CT-1 than CO in diabetic rats. However, the role of CT-1 in metabolism, obesity, and hyperglycemia in T2DM have been identified [32]. Nevertheless, the underlying biochemical/molecular mechanisms still need to be illuminated.

## 5. Conclusion

The current work investigates the effect of HIIT and CO protocols on apelin/APJ, NO, and cardiotrophin-1 in cardiac tissues in diabetic rats. We found that 8 weeks of HIIT and CO in rats with T2D induced similar adaptations on apelin, APJ, NO, and cardiotrophin-1 in cardiac tissue. Moreover, the results also show the beneficial impacts of exercise in diabetics, despite its mode. However, strong supporting research is further needed.

## Figures and Tables

**Table 1:** Bodyweight, serum glucose, and weight of animals in different rat groups.

Group	Preweight (kg)	Postweight (kg)	Preglucose (mg/dl)	Postglucose (mg/dl)
C-0	239 ± 26.7	305 ± 14.44	377.6 ± 40.41	334.19 ± 27.94
C-2	233 ± 24.78	295.2 ± 18.26	369.6 ± 39.04	350.13 ± 18.75
HIIT-0	237.5 ± 21.22	205.6 ± 8.26	319.3 ± 34.45	300.8 ± 12.98
HIIT-2	236.3 ± 26.5	207.5 ± 16.52	330.6 ± 35.97	297.18 ± 22.15
COT-0	223.83 ± 29.57	212 ± 13.75	348.16 ± 27.43	305.19 ± 31.85
CO-2	229.83 ± 18.73	214.86 ± 12.45	360.16 ± 36.33	315.17 ± 18.95

## Data Availability

The data used to support the findings of this study are available from the corresponding author upon request.
